# Integrating electrodermal biofeedback into pharmacologic treatment of grand mal seizures

**DOI:** 10.3389/fnhum.2015.00252

**Published:** 2015-05-11

**Authors:** Tullio Scrimali, Damiana Tomasello, Massimo Sciuto

**Affiliations:** ^1^Department of Psychiatry, Medical School, University of CataniaCatania, Italy; ^2^ALETEIA Clinical CenterCatania, Italy

**Keywords:** biofeedback, grand-mal seizures, electrodermal activity, sympathetic arousal, epilepsy

## Abstract

Electrodermal activity (EDA) and electrodermal biofeedback, when integrated with pharmacologic treatments, indicate promising methods for the treatment of grand mal seizures. They can be used to monitor patient arousal and help patients learn new strategies to better cope with stress and anxiety. Our proposed method can possibly reduce the number of crises for patients who are dependent on pharmacologic therapy and can improve their quality of life. This article describes the scientific background of electrodermal monitoring and electrodermal biofeedback for patients affected by grand mal seizures. In this study, we have reported a clinical case study. The patient was treated for 2 years with electrodermal biofeedback to augment pharmacologic treatments. The trial has been designed in accordance with “*n = 1 case study research*”. Our results have shown that our methods could achieve a significant reduction in grand mal seizures and sympathetic arousal when applied. The patient under consideration was also relaxed and exhibited greater competency to cope with stress. Additionally, the patient’s sense of mastery and self-efficacy was enhanced.

## Introduction

Electrodermal activity (EDA) is a biological parameter that offers information about the psychological condition of an individual (Prokasy and Raskin, 1973). It reflects the level of functioning of sweat glands that are linked to the dynamic processes of the central and peripheral nervous systems (Scrimali, 2012).

The human brain has many different modules and cortical areas, involved in EDA control, but the data currently remains incomplete (Boucsein, 1992). Table 1 depicts the general picture today (Wang, 1964; Sequeira and Roy, 1993).

**Table 1:** **Level and its corresponding module(s) and involved area(s)**.

Level	Modules and Areas Involved
Cortical	• Sensorimotor area
	• Precentral motor cortex
	• Brodmann area 6 of the temporal lobe
Striatal	• Anterior limbic and infralimbic cortex
	• Some parts of pallidum
	• Caudate nucleus
Diencephalic	• Dorsal thalamus
	• Anterior hypothalamus
Mesencephalic	• Lateral reticular formation of the midbrain
Rhomboencephalic	• Roof nuclei of the cerebellum
	• Ventromedial reticular formation of the midbrain
Spinal	• A pool of sympathetic motoneurons controlling sweating secretion in the spinal cord
Peripheral	• A ganglion of the sympathetic chain

Generally, EDA is measured by applying two electrodes to the palmar surface of the fingertips. We also describe some alternate electrode placement for recording EDA (Boucsein, 1992) and, further, more alternate methods for acquiring electrodermal data are reported. These alternate methods are as follows: endosomatic recording without the application of an external current, exosomatic recording with direct current, and exosomatic recording with alternating current (Boucsein et al., 2012).

We have chosen placements that involve the volar surfaces of the distal phalanges. The apparatus we used, (MindLAB Set by Psychotech) produces a small direct electrical current, (constant voltage) between the two electrodes, that indicates EDA. EDA is considered as any change in the conductance produced by any variation in sweat from an individual. In this case, *exosomatic direct current* and* constant voltage method* have been adopted (Boucsein et al., 2012).

The potential is recorded between an area that is characterized by many sweat glands and other areas that are characterized by few sweat glands. This is known as an endosomatic measurement. Both exosomatic and endosomatic activities are caused by sweat, which is a salt solution. An increase in the levels of sweat for an individual is indicated by an increase in the ions present on the skin. The presence of many ions can increase the electric conductance of the skin (Dawson et al., 2007).

In the field, generally speaking, exosomatic measurements are used because they are easier to register. Currently, exosomatic EDA is a well-documented psychophysiological parameter that provides information about arousal, stress, and clinical anxiety for various psychosomatic and psychiatric disorders (Prokasy and Raskin, 1973).

Biofeedback uses an electronic apparatus to monitor (online) the changes of some physiological functions in order to modify and control these functions. The biofeedback settings represent an individual’s interaction with the instrumentation (Khazan, 2013). Other variables are also included in the loop such as the therapist, the environment, and the cognitive activities of the patient. Biofeedback includes the following components: (1) physiological parameters being measured; (2) electrodes; (3) a transducer; (4) a differential amplifier; (5) an analysis unit that can process biological signals; and (6) a display unit that can produce auditory and visual feedback related to the measured parameters. We have distinguished two types of visual display: analog and digital.

Grand mal seizures are characterized by increased sympathetic arousal (Nagai et al., 2004a). Therefore, biofeedback for EDA is aimed at the reduction of this arousal, which can be useful as part of an integrated approach for the treatment of grand-mal seizures (Boon et al., 2001; Nagai et al., 2004b; Porges, 2011; Nagai and Trimble, 2014).

## Materials and Method

We have conducted a *single clinical case study* in accordance with the methodological principles adopted in *n = 1 case study research* (McLeod, 2010).

A 33-year-old male patient who has been treated for 2 years with electrodermal biofeedback and the number of crises has been carefully monitored.

We carried out our research according to the laws and regulations of our Country (Italy) as well as according to any applicable international norms and standards, mainly the Helsinki Declaration (World Medical Association, 2012). The patient signed an informed consent form to participate in our study. Our protocol was preliminarily revised and accepted by the neurological staff of the Institution in charge of curing the patient (a public Hospital). Neurologists recognized that the proposed research cannot damage in any way the patient and, on the contrary, that the use of electrodermal biofeedback could be a positive change for bettering his clinical condition.

The patient suffers from grand mal seizures and has been treated with various antiepileptic drugs since he was 7 years old. The diagnosis was confirmed using electroencephalographic (EEG) evidence and brain mapping. The grand mal seizures were referred to as a large malformation of the brain located on the cortex of the right hemisphere of the patient. Since the malformed area was too large, a treatment based on surgery was not considered by neurosurgeons. In spite of his adherence to pharmacologic treatment, the patient suffered from crises during periods of increased stress and sympathetic arousal. The patient reported that seizures were linked with anxiety, interpersonal stress, and before a crisis, a headache was frequently present. The patient used two medications: carbamazepine (600 mg, twice a day) and lacosamide (200 mg, twice a day).

The patient was treated, before our research, for many years, with various and different protocols based on different medications. The protocol that was used during our research also showed the best results. For this reason, the Neurological staff decided that it was not necessary to change medications for the patient during our trial. The patient was treated at an outpatient applied neuroscience unit that was set up in a private clinical center known as the *Centro Clinico ALETEIA*.

We informed the patient that the treatment was experimental and for this reason, we offered care for free. The patient came each week for a 1 h sessions for a period of 2 years. During these sessions, EDA as well as biofeedback trials were monitored. In addition, problem-solving and coping strategies based on cognitive and behavioral approaches were used.

The MindLAB Set system, produced by Psychotech, was used. The set is composed of the hardware (Psychodata Acquisition Unit), a pair of electrodes, and an interface device for connection to a computer. Two integrated programs, MindSCAN and Psychofeedback, were used to monitor and record EDA (MindSCAN) as well as to conduct the weekly biofeedback sessions (Psychofeedback).

For each session, the patient was asked to sit in a comfortable *Chaise Longue*. The temperature in the laboratory was frequently monitored and maintained at 22°C in winter and 24°C during summer. A monitor and two speakers were positioned in front of the patient for providing visual and acoustic feedback connected with the Skin Conductance Level (SCL).

The patient was encouraged to “lower the arousal” by acting on thinking, imagery, emotion, and somatic levels. The patient was further encouraged to establish different kinds of cognitive, emotional, and bodily-different attitudes. When the graph of SCL was being drawn on the monitor and reinforcement sounds were heard, the patient was encouraged to understand and memorize the specific set realized in that specific moment in his mind and body. On the contrary, when both visual and acoustic feedback informed the patient that the arousal was going up, he was trained to understand the kind of negative attitude he was producing and then to avoid it.

The patient developed an understanding and realization of the coping strategy that are useful to reduce arousal. Then he was encouraged to generalize this to real life conditions. At the beginning of each session, before practicing biofeedback, SCL was measured and the recorded values were discussed together with the therapist. When SCL was higher, i.e., when it indicated a warning sign of stress, the patient was encouraged to find the reason for it and was encouraged to try to reduce the stress. The training was intuitive, comfortable, and interesting for the patient.

## Results

**Figure 1:**
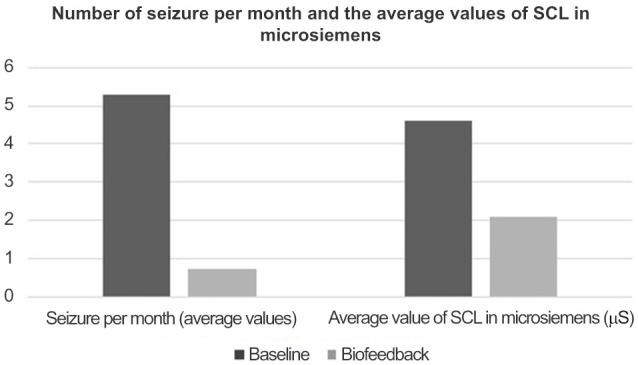
**Results of research**.

The average number of seizures over a 3-month period prior to biofeedback training and cognitive and behavioral therapy was 5.3. This reported frequency had been essentially stable during the last 6 months before the reported baseline (Figure 1).

During the following 2 years in which the patient took part in weekly biofeedback treatments, the average number of seizures was reduced to 0.75. The average SCL was 4.5 microsiemens during the baseline period and 2.1 microsiemens during the biofeedback treatment period (Table 2).

**Table 2:** **Number of seizures per month and the average values of SCL (in microsiemens)**.

	Baseline	Biofeedback
Seizures per month (average values)	5.3	0.75
Average value of SCL (in microsiemens)	4.6 μS	2.1 μS

Table 2 shows the data that has been statistically analyzed using Student *t*-tests. From our analysis, we found that *t* = 4.636 and *p* = 0.044. Therefore, the differences observed between the values of the two studied parameters (the number of seizures and the value of SCL expressed in microsiemens) measured before and during the treatment are statistically significant.

## Discussion

Our treatment, based on electrodermal biofeedback and applied from the use of the MindLAB Set system that was integrated into the pre-existing medication has indicated better outcomes than just for the pharmacologic treatment for grand-mal seizures. Since not much literature exists on seizure treatments that integrate biofeedback with traditional pharmacologic treatment, it was difficult to analyze our findings. Micoulaud-Franchi et al. (2014) have underlined that the neurofeedback protocol on sensorimotor rhythms (SMR) has been investigated in many studies, while newer neurofeedback protocols on slow cortical potential (SCP) and EDA-biofeedback protocols have been used for just a few studies (Micoulaud-Franchi et al., 2014).

The results of our research show that EDA biofeedback can improve the treatment profile of people affected by grand mal seizures. Moreover, the number of crisis per month, which is the most clinical item, when treating seizures diminished significantly.

Recently, Nagai (2014) suggested that seizure precipitation could occur due to certain psychological factors such as stress, anxiety, and depression. Our results show that EDA-biofeedback can reduce arousal and improve mood. According to Nagai (2014), EDA-biofeedback can improve thalamo-cortical regulation of neural excitability across brain networks as it focuses on the reduction of peripheral sympathetic tone.

The patient who participated in our study was more relaxed and exhibited a greater competency in coping with stress. In addition, the patient’s sense of mastery and self-efficacy was enhanced. This observation during the patient’s treatment could be an interesting future topic; however, this has not been systematically investigated by using any specific assessment instruments. Thus, further investigations are required to examine how biofeedback, when successfully integrated with pharmacologic treatments of grand mal seizures can improve important psychological variables such as mastery, self-efficacy, and self-esteem.

In conclusion, the use of EDA-biofeedback seems to be an interesting treatment procedure compared to Neurofeedback, with respect to costs and possibilities of disseminating the treatment clinically. EDA-biofeedback is inexpensive and can be put into practice without any specific training. In terms of the cost of professional devices for Neurofeedback and EDA-biofeedback, the latter can be integrated into the treatment of grand mal seizures before trying Neurofeedback, which is specific and expensive.

## Conflict of Interest Statement

The authors declare that the research was conducted in the absence of any commercial or financial relationships that could be construed as a potential conflict of interest.
